# Dr. Max A. Keiser

**Published:** 1913-01

**Authors:** 


Dr. Max A. Keiser, Niagara, 1894, of Buffalo, died Dec. 10, aged 50. His death is ascribed to eating tainted food when returning from a study trip to Europe. He was a laryngologist of note, having given more than usual attention to otology.

